# Stress Detection Using Frequency Spectrum Analysis of Wrist-Measured Electrodermal Activity

**DOI:** 10.3390/s23020963

**Published:** 2023-01-14

**Authors:** Žiga Stržinar, Araceli Sanchis, Agapito Ledezma, Oscar Sipele, Boštjan Pregelj, Igor Škrjanc

**Affiliations:** 1 “Jožef Stefan” Institute, Jamova cesta 39, 1000 Ljubljana, Slovenia; 2 Faculty of Electrical Engineering, University of Ljubljana, Tržaška cesta 25, 1000 Ljubljana, Slovenia; 3 Computer Science Department, University Carlos III of Madrid, 28911 Leganés, Madrid, Spain

**Keywords:** affective computing, EDA, stress detection, physiological signals, frequency analysis

## Abstract

The article deals with the detection of stress using the electrodermal activity (EDA) signal measured at the wrist. We present an approach for feature extraction from EDA. The approach uses frequency spectrum analysis in multiple frequency bands. We evaluate the proposed approach using the 4 Hz EDA signal measured at the wrist in the publicly available Wearable Stress and Affect Detection (WESAD) dataset. Seven existing approaches to stress detection using EDA signals measured by wrist-worn sensors are analysed and the reported results are compared with ours. The proposed approach represents an improvement in accuracy over the other techniques studied. Moreover, we focus on time to detection (TTD) and show that our approach is able to outperform competing techniques, with fewer data points. The proposed feature extraction is computationally inexpensive, thus the presented approach is suitable for use in real-world wearable applications where both short response times and high detection performance are important. We report both binary (stress vs. no stress) as well as three-class (baseline/stress/amusement) results.

## 1. Introduction

Affect recognition is an interdisciplinary field that touches on signal processing, machine learning, psychology and physiology. Affective state recognition is useful in a number of scenarios. One affective state that has received a lot of attention recently is stress [1,2,3,4,5,6], especially since it has been demonstrated that frequent and prolonged stress has negative effects on mental and physical well-being, immune system, lower employee efficiency, lower employee engagement, more sick days, etc. [1,7,8].

The field of affect recognition aims to identify and label different affective states. Since the affective state of a subject is a psychological state inherent to the observed subject, it is not directly observable. Therefore, affect recognition uses several other observable variables to achieve its goal. These include (1) physiological signals such as electrocardiogram (ECG), EDA, electromyography (EMG), respiratory rate and subject temperature [5,9,10,11]; (2) subject context data such as accelerometer data, behaviour patterns, GPS data and smartphone usage [12]; (3) alternative subject data such as video or audio recordings [11]; (4) environmental contexts such as ambient temperature and humidity [13]

Recent advances in wearable technology [6,14] have led to increased research interest in wearables-focused affect recognition, e.g., [4,5,6,12,15,16]. With the increasing popularity of wearable electronics, algorithms that aim to detect affect using wearable sensors have a greater chance of being used in practice.

In real-world applications, two aspects of any proposed solution are important: (1) detection performance and (2) algorithm responsiveness—the time from onset of the affective state to detection (time to detection—TTD). Our goal is to present an approach to stress detection that provides competitive performance while improving responsiveness. In Section 1.1, Section 1.2, Section 1.3 and Section 1.4, we provide background information on affective state detection with wearable sensors. In Section 2, we first show some recent results on stress detection using wearable EDA sensors and then present and explain our proposed method. Results and discussion can be found in Section 3.

### 1.1. WESAD

Several datasets on affect recognition are publicly available, for example, DEAP [9], NonEEG [10], WESAD [5] and MAHNOB-HCI [11]. While some datasets work in valence-arousal space (Russel’s circumplex model [17]), WESAD works with the categorical model. It is more focused on stress than some of the other datasets; the main purpose of the dataset is stress detection. WESAD also focuses on wearable sensors, making it perfect for our needs. The multimodal dataset includes both chest and wrist-based measurements of acceleration, ECG, BVP, EDA, EMG, respiration and temperature. The dataset includes fifteen subjects and three major affective states: baseline, stress and amusement.

Some advantages of the WESAD dataset are:

1. Chest and wrist measurement: the same type of physiological signals are measured at two sites, allowing comparison and validation
2. Real-world tasks: The tasks that the subjects performed are likely to occur in real life, such as public speaking or job interviews. Although WESAD was recorded in a laboratory environment, the dataset is comparable in this respect to data generated in the field.
3. High sampling frequency: data collected with a chest-worn device were sampled at 700 Hz; data from the wrist-worn Empatica E4 (http://www.empatica.com/research/e4/, accessed on: 20 November 2022) were sampled at lower frequencies, up to 64 Hz.
4. The authors present a reference classification approach and give a baseline accuracy and F1 scores.

A number of stress detection methods have been evaluated using WESAD. Their results are given in Section 2.1.

### 1.2. EDA

EDA broadly refers to any change in the electrical properties of the skin’ Greco et al. [18], skin conductance (SC) is a commonly used measure of EDA. Activation of the sweat glands by the autonomic nervous system (ANS) is reflected in SC.

Skin conductance is usually interpreted as an aggregation of two components: the slower ‘tonic’ (SCL—skin conductance level, EDL—electrodermal level) and the faster ‘phasic’ (SCR—skin conductance response, EDR—electrodermal response). The latter is interpreted as a short-term reaction to stimuli and is, therefore, of particular interest in affect recognition. In [18], the disaggregation problem is formulated as a convex optimization problem and a solution is provided.

In [15], the drawbacks of using EDA in wearable stress detection research are pointed out. The basis of the critique is that EDA was not supported in the most widely used wearable operating systems at the time. For example, in [5] the EDA signal is measured at the wrist with the Empatica E4, which is not actually a consumer-oriented product. However, since the article was published, new smartwatches have hit the market, some of which include an EDA sensor (Fitbit Charge 5, Fitbit Sense). Furthermore, a patent filed by Apple [14] indicates the possible future inclusion of EDA sensors in the smartwatch product range in the future. Therefore, we believe that the use of EDA for stress detection in wearables should be explored and appropriate methods developed.

### 1.3. EDA Feature Extraction

In [19], three EDA-based features for stress detection are presented. The features are also used in [5,15]. The three features are:

(1) $$\begin{array}{cc} \mu_{SCL}= & \frac{1}{N}\sum_{i=1}^{N}{\hat{X}}_{SCL}\left(t-i\right) \end{array}$$

(2) $$\begin{array}{cc} \rho_{SCL}= & \rho (t,{\hat{X}}_{SCL}) \end{array}$$

(3) $$\begin{array}{cc} \sigma_{SCR}= & {\left(\frac{1}{N}\sum_{i=1}^{N}{\hat{X}}_{SCR}^{2}\left(t-i\right)\right)}^{1/2} \end{array}$$

$\mu_{SCL}$ is the average SCL trend over the last *N* samples, $\rho_{SCL}$ captures the degree of linearity of SCL and $\sigma_{SCR}$ captures the standard deviation in SCR.

Four skin conductance features are defined in [20]. The four features are calculated from the absolute value of skin conductance by determining and analysing the local minima and maxima of the measurements. The four features are calculated for a sliding window and give the number of peaks, the sum of the peak values, the sum of the minima values and the estimated area under the response of the skin conductance. The features are also used in [5,15].

The FLIRT toolkit [21] is an open-source Python package for data processing and feature extraction for wearable sensor data. According to the authors, the contribution of the package lies in its holistic approach—the package includes methods for loading datasets, preprocessing, segmentation and feature extraction. The package focuses on four signals: ECG, IBI (inter-beat interval), EDA and ACC (accelerometer).

The EDA features included in FLIRT are statistical, entropy-based, time domain, frequency domain, time-frequency domain and SCR peak-based features:

- time domain: 29 SCR and 21 SCL features are calculated, including statistical (mean, standard deviation, minimum, maximum, percentile values, interquartile range), energy, entropy, number of peaks, SCR peak properties, etc.
- frequency domain: 10 SCR and 10 SCL features including energy, interquartile range, variance and spectral powers at specific frequencies.
- time-frequency domain: 6 statistical features for each EDA component, 12 in total.

Another Python toolkit for EDA feature extraction is available—pyEDA [22]. The authors propose statistical features based on peak detection and raw RDA signal. The authors also propose the use of an autoencoder for feature extraction. The autoencoder is an unsupervised artificial neural network. A total of 64 features are obtained by the authors in this way.

The EDA signal, like any other measured physical signal, is continuous. Most data processing pipelines and algorithms work with data vectors of limited length, often of a fixed length. Therefore, the continuous EDA signal must be segmented and converted into fixed-length segments. The commonly used approach is the sliding window. In sliding window segmentation, the window length and the segmentation step are the two parameters. Many researchers do not give much thought to these, in EDA analysis, 60 s windows are the most commonly used. In [15], different window lengths are analysed for feature extraction. It is reported that longer windows lead to better stress detection, with the longest window studied, 120 s, performing better than the others.

### 1.4. Evaluation of Stress Detection

The goal of affect recognition and stress detection is to develop algorithms for use in the real world. The generalization property of an algorithm refers to its ability to perform *well* on unseen data. In the case of affect recognition, *unseen* can mean a new user who was not present during training. To evaluate an algorithm for this scenario, the leave one subject out (LOSO) cross-validation approach is used.

LOSO requires that the training dataset comes from a set of subjects. The dataset must also be appropriately labelled. For *J* subjects, *J* models *M* are trained: *M_j_*, 1≤j≤J. The *j*-th model is trained on training data without the data of subject *j*. Subject *j*’s data are only used for model validation. In this way, *J* models are trained and scored to produce *J* scores. The scores are then averaged to determine the total score of the algorithm.

The most common score for classifiers is the accuracy score (4). It is calculated based on the number of true positives (TP), false positives (FP), true negatives (TN) and false negatives (FN).

However, for unbalanced datasets, the F1 score (5) is usually used in such scenarios. WESAD is unbalanced.

(4) $$Accuracy=\frac{TP+TN}{TP+TN+FP+FN}$$

(5) $$F1=\frac{2TP}{2TP+FP+FN}$$

## 2. Materials and Methods

In the following sections, we give an overview of the state of the art in EDA-based stress detection with wearable sensors (Section 2.1), describe segmentation and frequency analysis (Section 2.2), and then present our approach in Section 2.3, Section 2.4, Section 2.5 and Section 2.6.

### 2.1. Stress Detection Using EDA from Wearable Sensors

Several authors have used WESAD to evaluate their stress detection algorithms. This section lists the results obtained by these authors.

#### 2.1.1. Reference WESAD Classification Results

In WESAD [5], the authors provide reference classification results. Binary (stress vs. no stress) and three-class classification results (stress vs. baseline vs. amusement) are provided. As this is a multimodal dataset (10 sensors), each sensor is used both independently and in groups (all wrist-based, all chest-based, etc.). Five classifiers are evaluated (decision tree, random forest, AdaBoost with decision tree, linear discriminant analysis and K-nearest neighbours). For each, both the F1 score and the accuracy are reported.

The best results for three-class classification were obtained with chest-based ECG, EDA, EMG, respiratory rate and temperature data. The best classifiers for three-class classification were AdaBoost and linear discriminant analysis for accuracy (80.34%) and F1 score (74.43%), respectively.

The best binary classification was obtained using chest-based ECG, EDA, EMG, respiratory and temperature data from the chest. The best classifier for both accuracy and F1 score was linear discriminant analysis, achieving 93.12% and 91.47%, respectively.

The best results of the WESAD authors focusing only on the wrist EDA signal are shown in Table 1. All other approaches aim to outperform this reference.

#### 2.1.2. Analysis by Siirtola

In [15], the same features are used as in [5]. The aim of the study is to investigate the effect of window size in the calculation of the features. The conclusion is that longer window sizes lead to better stress detection. The longest window size studied, 120 s, performs better than the other options (15 s and above). Three classifiers are used—LDA, QDA (linear and quadratic discriminant analysis, respectively) and RF (random forest). The best (binary) classification results (for WESAD) are obtained by LDA using skin temperature, BVP and HR signals—87.4%. F1 scores are not reported. The author uses the LOSO approach. Using EDA only, the author reports the best accuracy of 78.3% with the random forest classifier. No other hyperparameters are reported. All results were obtained using a 120 s window, which the authors consider optimal for their feature calculation and choice of classifier.

The reported accuracies are consistent with those of the WESAD authors.

#### 2.1.3. Deep Fusion Network

In [16], an explainable deep neural network approach to affect recognition is presented. The authors demonstrate their approach using the WESAD dataset. The model used is the ‘Multimodal-Multisensory Sequential Fusion’ (MMSF) model. The results show that the approach performs better than the benchmark dataset [5] in terms of both accuracy and F1 scores. The authors use a very short window—only one second. The authors use both chest and wrist modalities. Since they are dealing with a multimodal problem, signal fusion is required. After experimenting with early and late fusion, they decide to use late fusion, i.e., each modality trains its own model (neural network) and the results of all modalities are fused with an additional model (random forest). The reported results are an accuracy of 83% and an F1 score of 81% (in the three-class problem, using all chest modalities of WESAD). On further analysis, the authors found that EDA measured at the chest was the most important modality. In contrast, EDA measured at the wrist was found to be insignificant. This stark difference can be attributed to the different sampling rates. The EDA measured at the chest in WESAD is sampled at 700 Hz, whereas the Empatica E4, which is used to measure EDA at the wrist, samples at a much lower frequency. This results in too few measurements in the one-second window and thus poorer performance.

#### 2.1.4. StressNAS

In [3], deep neural networks (DNNs) are applied to the WESAD dataset. The proposed StressNAS is a data-driven approach for deep neural network design. The authors report a significant improvement in performance over the reference implementation in [5] and manual DNN design approaches. The results are presented in Table 2. The experiments were performed with a sliding window of 60 s width and 0.25 s step size.

The three-class classification accuracy obtained using StressNAS-designed DNNs (using the EDA signal) is 4.57% higher than the reference results.

#### 2.1.5. FLIRT Toolkit

FLIRT is a feature generation toolkit for wearable sensor data. The authors provide WESAD results [21]. They use 60 s windows with a step of 0.25 s, just as in [5]. The authors use LOSO cross-validation and the same classifiers as in [5] (LDA, RF, AB, DT). F1 scores for three class classifications are reported.

The three-class classification F1 score by the authors of FLIRT using EDA is reported as 51.96% (compared to 49.06% in [5]). Other values (binary, accuracy) are not given.

#### 2.1.6. XGBoost

The contributions of [23] to EDA stress detection are the use of the XGBoost classifier, an alternative segmentation approach, the use of a high number of features (almost 200) and the use of feature selection algorithms. The results related to improved segmentation are of interest as the authors found an improvement of about 15% over the reference (binary classification, F1 score, chest and wrist EDA, independently) just by using an alternative segmentation strategy. In [3,5,21], the authors used a 60 s sliding window with 0.25 s steps. In [23], the authors also used a 60 s sliding window but a 30 s step. (The authors report that they obtained only 1041 segments using this approach. Our calculations show that about 8000–9000 segments should be extracted).

In addition, the authors obtain relatively small improvements (a few percentage points) by using the XGBoost classifier and using (almost 200) additional features. The authors also propose a feature selection approach to reduce the number of features and thus improve the efficiency of the classification task without significantly degrading the performance.

The final result is 89.92% F1 score of the binary classification task when using the wrist-based EDA signal and 92.38% with the chest-based EDA.

#### 2.1.7. pyEDA

The authors of pyEDA [22], a Python library for EDA manipulation, also provide stress detection results for WESAD. However, the results reported by the authors cannot be compared with those of [3,5,15,16,21,23] because the authors do not use LOSO cross-validation and, more importantly, they define the binary classification problem as follows: ‘Baseline section labels as “not stressed” (0), and EDA data in the Stress section labeled as “stressed” (1)’, in this approach all data labelled as ‘Amusement’ are seemingly ignored.

The authors report an accuracy of 88–90% when only statistical characteristics are used. The accuracy increases to 95–97% when the autoencoder-generated features are used.

#### 2.1.8. Summary

The results of seven papers were listed. Of the seven, only the authors of the WESAD dataset gave F1 scores *and* accuracy for the binary *and* three-class problems (see Table 3). Most of the results were obtained without giving much importance to the choice of window size in the segmentation. The exception is [15], where an increase in accuracy is reported when the window size is increased. In our work, we focus on using the EDA signal measured at the wrist. Therefore, we only compare our results with EDA-based results from other authors. Deep fusion network results obtained using only EDA are not reported and, therefore, cannot be directly compared with our results.

Among the reported results, the deep fusion network, XGBoost and pyEDA stand out for their reported values. (1) For the deep fusion network, this improvement can be attributed to the use of more signals (all chest measurements). (2) pyEDA’s performance results are not directly comparable to the other results because they define the binary case differently than other authors. They do not report three-class classification. (3) The authors of XGBoost report the largest performance gain when using a thirty-second step in the segmentation. This result is surprising, but unfortunately, we could not replicate their results by combining their segmentation and our feature extraction.

### 2.2. Segmentation and Frequency Analysis

**Figure 1:**
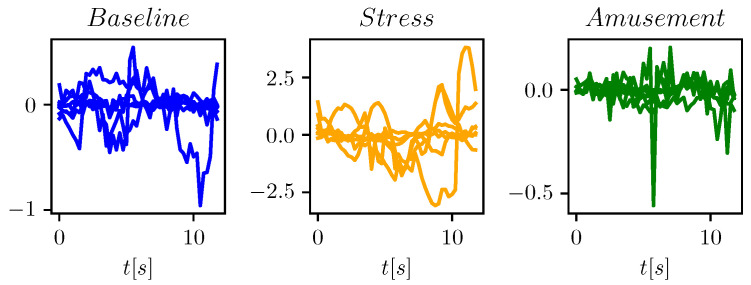
Examples of the three WESAD classes.

The original dataset contains continuous, non-segmented measurements. The dataset is segmented using the sliding window approach, just as in [5]. It is ensured that each segment contains only measurements related to a single affective state. The window width and step are configurable parameters whose choice we discuss later. Figure 1 shows examples of segments for each of the affective states in WESAD.

Much of the research focuses on temporal features, but we are exploring the use of frequency-based features. To obtain a frequency spectrum of a signal, the Fourier transform is performed. The transformation of the signal x(t) in the time domain to X(f) in the frequency domain is given in (6). For the discrete signal x(k), k∈N, the discrete Fourier transform (DFT) is used as given in (7).

(6) $$X\left(f\right)=\int_{-\infty }^{\infty }x\left(t\right)e^{-2\pi if}dt$$

(7) $$X\left(f\right)=\sum_{k=0}^{N-1}x\left(k\right)e^{-i2\pi fk/N}$$

For a non-periodic discrete signal x(k) of finite length 0≤k≤N−1, the DFT is discrete and periodic. For the analysis, we can restrict ourselves to the first period only and ignore the rest. Since the resulting DFT is an even function, the values at negative *f* may be ignored. We are left with $X=\{X_{1},...,X_{\frac{N}{2}}\}$. The frequency resolution of *X* is $\frac{1}{t_{s}N}$ ($t_{s}$ is the sampling interval of *x*, *N* is the number of samples).

The Fourier transform generally results in a complex signal. The signal consists of the absolute value (absolute spectra |X(f)|) and the phase (phase spectra ∠X(f)).

**Figure 2:**
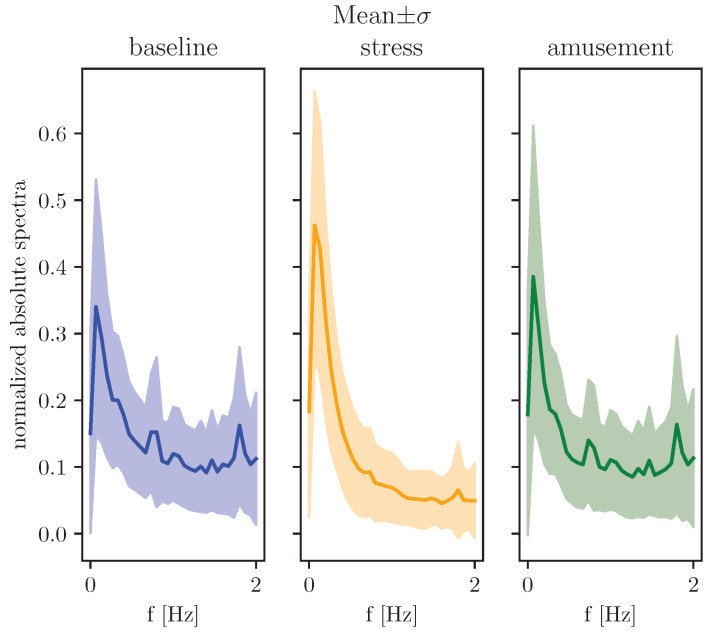
Normalized amplitude spectra of three classes from WESAD: mean and standard deviation.

We are interested in the DFT of the three affective states contained in WESAD. Each segment of the normalized dataset is labelled (stress, amusement, baseline). For each segment, DFT can be run and the absolute spectra computed. Figure 2 shows the power-normalized absolute spectra of the three classes. The mean and standard deviation envelopes are shown. It can be seen that the three classes differ in their amplitude spectra.

### 2.3. Feature Extraction

Following the time series analysis pipeline of (1) segmentation, (2) feature extraction and (3) classification, appropriate features should be extracted. Figure 2 suggests that the absolute spectra could be used to obtain features relevant to affect recognition. In the following sections, the features are introduced and explained.

#### 2.3.1. Frequency Band Selection

In Figure 2 the distinction between stress and the other two classes can be seen. A subset of the spectra (a frequency band) could be used to extract useful features. $|X^{b}|$ denotes the spectra *X* with amplitudes above and below the cut-off frequencies ($f_{low}$ and $f_{high}$) set to 0. We propose to divide the amplitude spectra into several frequency bands and calculate features for each band. Since the EDA signal measured at the wrist is sampled at 4 Hz, we propose two frequency bands: (1) $f_{low}=0.0$ Hz, $f_{high}=1.0$ Hz, (2) $f_{low}=1.0$ Hz, $f_{high}=2.0$ Hz. In this way, one set of features describes the low frequencies and a second set of features describes the higher frequencies.

(8) $$|X^{b}|\left(f\right)=\left\{\begin{array}{cc} 0, & \mathrm{if}\;f<f_{low}\\ 0, & \mathrm{if}\;f>f_{high}\\ |X|(f), & \mathrm{otherwise} \end{array}\right.$$

#### 2.3.2. Mean and Standard Deviation

For each amplitude spectrum $|X_{i,j,k}^{b}|\left(f\right)$ (class *i*, person *j*, exemplar *k*) the mean ${\bar{|X^{b}|}}_{i,j,k}$ and the standard deviation $\sigma_{i,j,k}\left(\right|X^{b}\left|\right)$ of the selected frequency band are calculated:

(9) $$N_{b}=\left(f_{high}-f_{low}\right)f_{s}$$

(10) $${\bar{|X^{b}|}}_{i,j,k}=\frac{1}{N_{b}}\sum_{f}\left|X_{i,j,k}^{b}\right|\left(f\right)$$

(11) $$\sigma_{i,j,k}\left(\right|X^{b}\left|\right)=\sqrt{\frac{1}{N_{b}-1}\sum_{f}{\left||X_{i,j,k}^{b}|\left(f\right)-\bar{|X_{i,j,k}^{b}|}\right|}^{2}}$$

#### 2.3.3. Max Amplitude Spectra

In addition to the mean and the standard deviation, other features can also be obtained. Figure 2 shows that the maximum value in highlighted frequency band can be used. (12) defines $\alpha_{i,j,k}^{b}$ as the maximum value of $|X_{i,j,k}\left(f\right)|$ in the selected frequency band.

(12) $$\alpha_{i,j,k}^{b}=\max_{f}\left|X_{i,j,k}^{b}\left(f\right)\right|$$

#### 2.3.4. Half-Energy Frequency

The amplitude spectra in Figure 2 show that higher frequencies are less present in the class ‘stress’ than in the other classes. This observation is exploited to obtain an additional feature.

According to the laws of signal processing, the square of the amplitude spectrum of a signal corresponds to the energy density spectrum of the signal—a description of the energy distribution of the signal over different frequencies [24]. The law is presented in (13). In (14) the normalized cumulative energy sum $Psi_{\int }\left(f\right)$ is introduced.

(13) $$\Psi \left(f\right)={\left|X\left(f\right)\right|}^{2}\;\to \;\Psi_{i,j,k}\left(f\right)={\left|X_{i,j,k}\left(f\right)\right|}^{2}$$

(14) $$\Psi_{\int }\left(f\right)=\frac{1}{\int_{0}^{\infty }\Psi \left(\omega \right)d\omega }\int_{0}^{f}\Psi \left(\omega \right)d\omega$$

The frequency $f_{P/2}$ that satisfies the Equation (15) is calculated and used as a new feature.

(15) $$\int_{0}^{f_{P/2}}\Psi \left(f\right)df=\int_{f_{P/2}}^{\infty }\Psi \left(f\right)df\;\to \;\Psi_{\int }\left(f_{P/2}\right)=0.5$$

This feature is calculated using the full amplitude spectrum and does not depend on the frequency band in (8).

### 2.4. Feature Space

**Figure 3:**
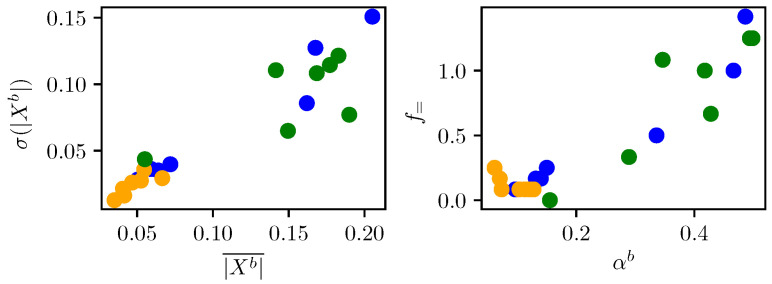
Seven exemplars from Figure 1 in feature space. Colours are analogous to Figure 2. The frequency band is set to $f_{low}=1.0$ Hz, $f_{high}=2.0$ Hz.

The exemplars from Figure 1 can be represented in the feature space. Figure 3 shows the features calculated for $f_{low}=1.0$ Hz, $f_{high}=2.0$ Hz. As expected, the stress samples (yellow) are partially separable from the other two classes.

### 2.5. Sequence Analysis

**Figure 4:**
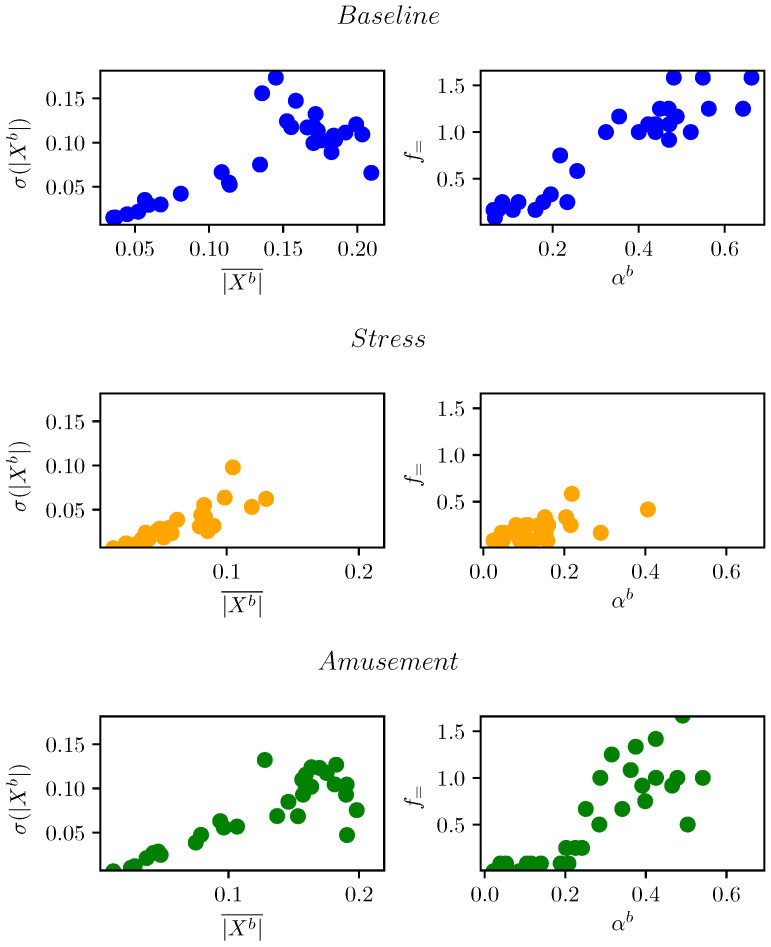
Thirty samples of each class from WESAD in feature space. Stress samples exhibit lower variance.

Figure 4 shows 30 randomly selected examples from each of the WESAD classes in the feature space. It shows that although the samples of baseline, stress and amusement may overlap in the feature space, the samples of stress have lower variance. In this section, we exploit this observation to obtain additional features.

To calculate the variance, an observation window *w* is defined. Then *w* successive samples are evaluated and mean and variance are calculated for each feature. This increases the number of features from 4 to 12 for each frequency band. Equations (16) and (17) show the calculations for feature $a_{i,j,m}$, where $a_{i,j,m}$ is one of ${\bar{|X^{b}|}}_{i,j,k}$, $\sigma_{i,j,k}\left(\right|X^{b}\left|\right)$, $\alpha_{i,j,k}^{b}$, $f_{P/2}$.

(16) $$M_{w}\left(a_{i,j,k}\right)=\frac{1}{w}\sum_{m=k-w}^{k}a_{i,j,m}$$

(17) $$V_{w}\left(a_{i,j,k}\right)=\frac{1}{w}\sum_{m=k-w}^{k}{\left(a_{i,j,m}-M_{w}\left(a_{i,j,k}\right)\right)}^{2}$$

### 2.6. Multiple Bands

The features derived in (10)–(12) are calculated for a single frequency band. However, multiple frequency bands can also be defined. For *m* frequency bands, a total of 3∗m+1 features are derived, since (8) is independent of frequency bands.

Applying (16) and (17) results in 3∗(3∗m+1) total features.

## 3. Results and Discussion

### 3.1. Evaluation

In Section 2.1, Section 2.2, Section 2.3, Section 2.4 and Section 2.5, we presented our approach to segmentation, analysis and feature extraction for stress detection using the EDA signal measured at the wrist. In this section, the results are presented, followed by a discussion of their relevance.

#### 3.1.1. Classifiers

The contribution of this work is the processing pipeline suitable for WESAD and the features derived in Section 2.3 and Section 2.5. To evaluate our contribution, a classification task is used. Different classifiers are used for classification, similar to [5,15,21,22,23].

Usually, classifiers are subjected to intensive hyperparameter tuning, but this is beyond the scope of this paper. Our goal is to show how the proposed pipeline and features can be used to achieve good performance in stress detection, rather than our ability to fine-tune a classifier. Therefore, our classifiers were not fine-tuned with respect to their hyperparameters. Moreover, we compare the results of the different classifiers used with the *best performing* classifier of the reference implementation in [5]. Therefore, an improvement in accuracy and F1 score would indicate that one of the classifiers using the presented pipeline and features presented, outperformed *all* classifiers used by Schmidt et al.

The following classifiers were selected for evaluation in our work:

- Decision tree, with a maximum depth of 10;
- 1-NN;
- kNN, where *k* (the number of neighbours) is set to 10;
- Random forest, where the number of trees is set to either 10 or 100, the maximum depth is 10 in both cases, and the class weights are balanced;
- Support vector machine (SVM) with radial kernel and balanced class weights;
- Bagged SVM;
- AdaBoost with random forest, with 10 estimators and each random forest consisting of 100 trees of depth 10 and balanced class weights.

For all the above classifiers, their implementations in Scikit-learn [25] (version 1.1.2) are used.

#### 3.1.2. Time to Detection

For practical stress detection, time to detection (TTD) is crucial. We define TTD as the difference between the timestamps of the first and the last sample used for classification. The time series used for training and evaluation were obtained using a sliding window approach with step $t_{step}$ and window width $t_{length}$. TTD is calculated as shown in Equation (18).

(18) $$TTD=t_{length}+\left(w-1\right)*t_{step}$$

### 3.2. Classification Results

Table 1 shows the results of the reference classification of Schmidt et al. [5]. The results were obtained using a LOSO approach and are presented for both the three-class and binary cases.

Table 4 shows the classification results obtained using the approach described in this paper. Segment length and overlap were set to $t_{length}=48*t_{s}$, $t_{overlap}=20*t_{s}$ ($t_{s}$ is the sampling time $t_{s}=\frac{1}{4\;\mathrm{Hz}}=0.25\;s$). To achieve a TTD of less than one minute, a window with w=7 segments was chosen. This results in a TTD of 54 s. A summary of the results:

1. For the binary case, *every* tested classifier, using the approach proposed by us, *outperforms, in terms of both accuracy and F1 score, the best performing* wrist-measured EDA classifier as reported by Schmidt et al.
2. For the three-class case, only the decision tree and the nearest neighbour classifiers lead to lower accuracy than the best-performing wrist-measured EDA classifier using the established EDA features. In all other cases tested, the proposed approach performs better than the established features.

### 3.3. Constant TTD

In Section 3.2, a single $t_{length},t_{step},w$ combination was evaluated. We are interested in a more comprehensive evaluation of the method as well as a comparison of a range of possible values.

**Figure 5:**
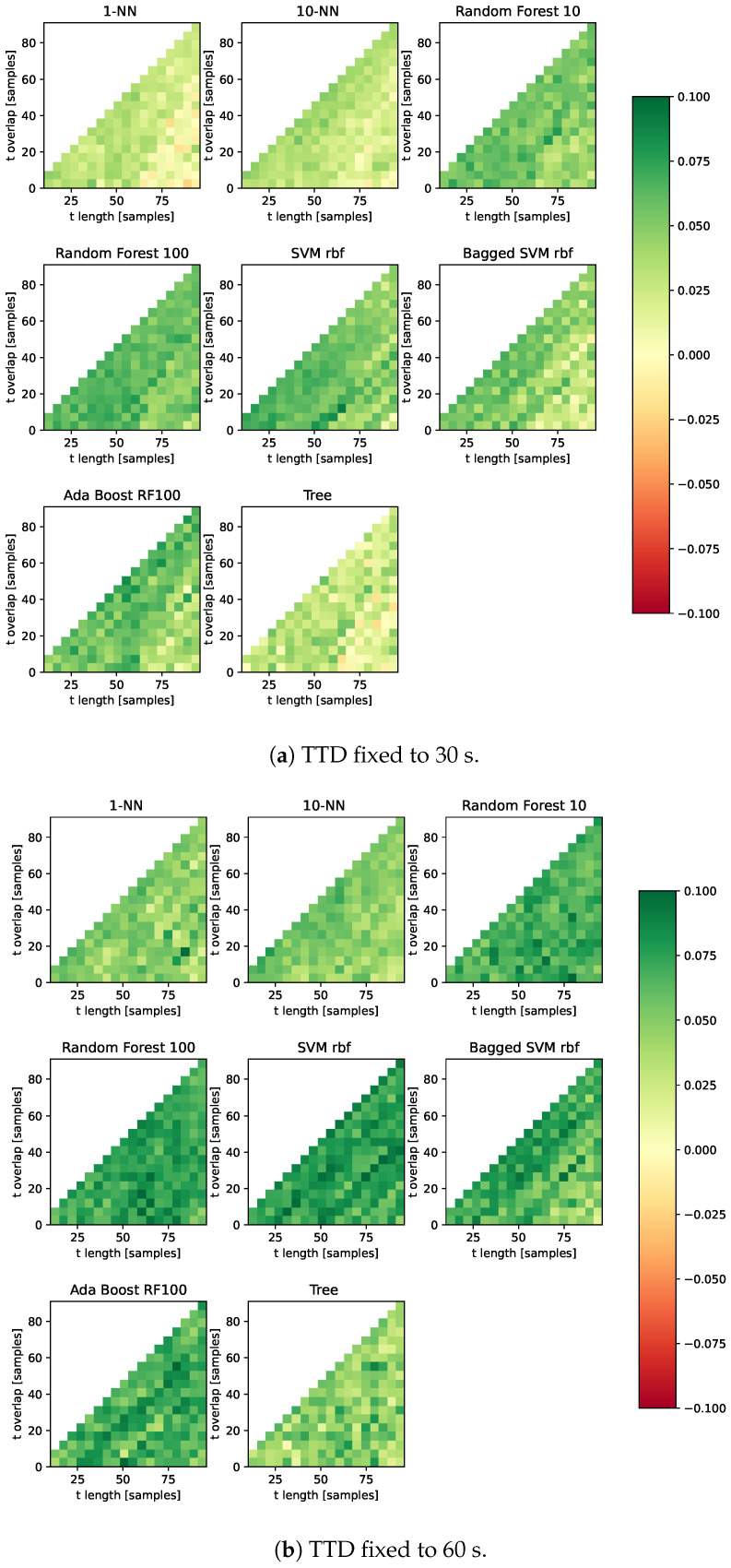
Improvement over Schmidt et al. (green = better), F1 score, three classes (baseline, stress, amusement).

$t_{length}$, $t_{overlap}$, *w* and TTD are linked in Equation (18). For a fixed TTD, *w* can be determined for each $t_{length}$ and $t_{overlap}$. In this section, the TTD was set to either 30 s (Figure 5a) or 60 s (Figure 5b). $t_{length}$ and $t_{overlap}$ were iterated in a grid search (requiring $t_{overlap}<t_{length}$). At each combination, *w* was calculated (which requires w∈N). Once *w*, $t_{length}$ and $t_{overlap}$ are known, a dataset can be created and a classifier trained and evaluated. Figure 5a,b show the comparison of the classifiers with the best results of Schmidt et al.

A comparison of Figure 5a,b shows a significant drop in performance when the TTD is lowered. This is understandable as the classifier works with data that spans a shorter period of time (30 s versus 60 s).

### 3.4. Effect of Window Size

Figure 5a,b show the importance of choosing the correct window size. The effect of window size in stress detection has been studied before, for example in [15]. In this section, we investigate the effect of window size on classification results using our proposed features.

**Figure 6:**
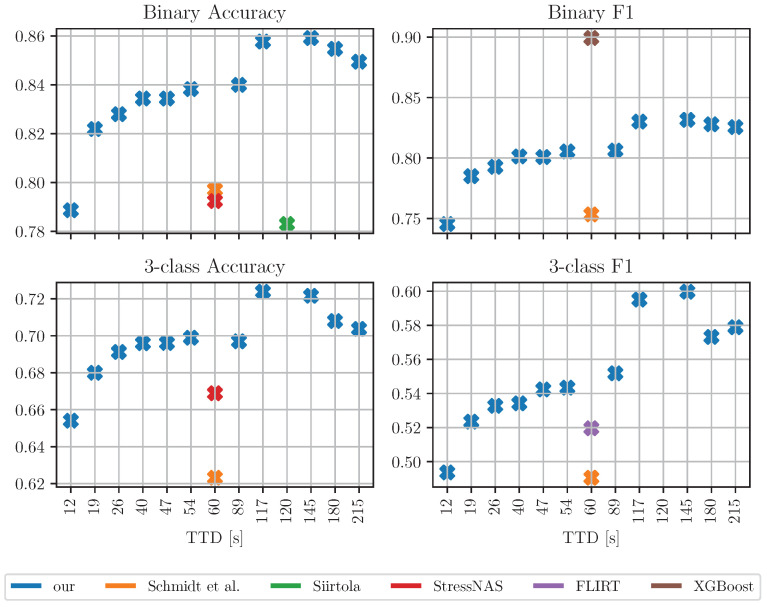
Effect of window size. $t_{length}=48,t_{overlap}=20$. Blue crosses: our results at various times to detection (x-axis (s)), using kNN classifier (k = 10). Orange, green, red, purple and brown crosses: results by other authors. Siirtola uses 120 s segments, while others authors use 60 s. See Table 3 for further details on results from other authors.

In Figure 6, $t_{length}=48,t_{overlap}=20$ are used and different window sizes are evaluated. For each calculated TTD, the accuracy and F1 scores are calculated for both the binary and three-class cases. The aim of this experiment is to show how changes in TTD affect detection performance. In this experiment, only one classifier was used, the simple kNN (k=10) classifier. The classifier was chosen because of its high three-class accuracy in Table 4.

Figure 6 shows a positive trend with increasing TTD. Therefore, a balance between performance and short TTD must be found when choosing the window size. The figures also show the results of Schmidt et al., Siirtola, StressNAS, FLIRT and XGBoost. The figure shows that the results of the other authors were obtained with window sizes of 60 s or more. The approach we present outperforms the other approaches, even when it provides faster stress detection (shown as lower TTD in Figure 6)! Moreover, we use the simple kNN classifier, while some of the other authors use much more complex classifiers.

### 3.5. Discussion of Results

In Section 3.2, Section 3.3 and Section 3.4, we have demonstrated considerable improvements over the results of other authors. We focused only on the EDA signal measured at the wrist. The results show that the features defined in Section 2.3 provide improved classification performance.

Most other authors have used a 60 s window to obtain their features, Siirtola uses a 120 s window. We have investigated other window widths and have shown that our method performs well with shorter windows reducing the time from the onset of stress to first detection (TTD). As the window increases, the performance improves.

It is worth noting that for all other authors, we used their *best* results with the wrist-measured EDA in WESAD, even so, the proposed method clearly outperformed them on many occasions.

## 4. Conclusions

Affective state recognition is an active area of research, and with advances in wearable technology, recognition using wearable sensors is becoming increasingly important. Among affective states, stress has been extensively researched, in part because of its impact on productivity and well-being.

This paper explores the use of frequency analysis of EDA signals obtained from wearable sensors for stress detection.

The main contribution of this paper is the proposed method for extracting features in the frequency domain. Throughout the article, the idea is explored and validated several times. The approach is validated using data from the publicly available WESAD dataset for stress detection by wearable sensors. Our results are compared with existing techniques and those published by several other authors. The proposed approach improves the accuracy and F1 scores of some existing techniques for stress detection from (wearable measured) EDA signals.

For any detection algorithm (fault, stress, etc.), the time from onset to detection is an important performance metric in addition to the accuracy of detection. The proposed approach allows for a customizable window length and thus a large number of possible values for time to detection (TTD). A trade-off between a short detection time and accuracy has been demonstrated. The proposed method has been shown to provide higher detection accuracy with a lower TTD than other comparable methods.

The work presented in our paper is an improvement over some of the commonly used methods and can be used in practical applications for stress detection.

## Figures and Tables

**Table 1:** Reference WESAD classification scores [5] for stress detection from wrist-based EDA measurements. AB: AdaBoost with decision trees; LDA: linear discriminant analysis.

	Best F1-Score (%)	Best Accuracy (%)
	(Classifier)	(Classifier)
**binary**	75.34±0.57 (AB)	79.71±0.43 (AB)
**three-class**	49.06±0.59 (AB)	62.32 (LDA)

**Table 2:** StressNAS: Accuracy of stress detection (WESAD). All wrist signals: BVP, EDA and temperature. (Δ [5]): an improvement over reference implementation.

	All Wrist Signals (Accuracy %)	EDA (Δ [5]) (Accuracy %)
**Stress vs. No stress**	93.14	79.24 (−0.47)
**Three-class**	83.43	66.89 (+4.57)

**Table 3:** WESAD results as reported by other authors. ACC: accuracy. * The authors use a 30 s step with the sliding window, this results in prolonged time from stress onset to detection (60 s window + up to 30 s waiting period, in total up to 90 s). ** The results are with chest-measured signals (results using only wrist-measured EDA were not reported).

Approach	2 Class ACC (%)	2 Class F1 (%)	3 Class ACC (%)	3 Class F1 (%)	Window [s]
Siirtola [15]	78.3	-	-	-	120
DFN [16] **	-	-	83	81	1
StressNAS [3]	79.24	-	66.89	-	60
FLIRT [21]	-	-	-	51.96	60
XGBoost [23]	-	89.92	-	-	60 *

**Table 4:** Classification results of our proposed approach, and comparison to best results achieved by Schmidt et al. [5]. Δ is the absolute difference in accuracy or F1 score. Positive values of Δ indicate better performance by our approach. NNL nearest neighbour; RF: random forest; SVM: support vector machine.

	Binary Case			
	Accuracy (%)		F1 (%)	
	Score	Δ [5]	Score	Δ [5]
Decision Tree	84.15	4.44	81.58	6.24
1NN	81.95	2.24	78.58	3.24
10NN	83.82	4.11	80.54	5.20
RF 10	84.87	5.16	82.13	6.79
RF 100	84.84	5.13	81.82	6.48
SVM	82.69	2.98	80.08	4.74
Bagged SVM	82.12	2.41	79.93	4.59
AdaBoost RF 100	83.86	4.15	80.29	4.95
	**Three-Class Case**			
	**Accuracy (%)**		**F1 (%)**	
	**Score**	**Δ [5]**	**Score**	**Δ [5]**
Decision Tree	58.53	−3.79	52.68	4.62
1NN	62.21	−0.11	53.74	4.68
10NN	69.89	7.57	54.34	5.28
RF 10	65.75	3.43	56.08	7.02
RF 100	66.86	4.54	56.36	7.30
SVM	67.08	4.76	56.78	7.14
Bagged SVM	67.56	5.24	56.20	7.14
AdaBoost RF100	67.00	4.68	54.67	5.61

## Data Availability

The data presented in this study are openly available in the WESAD dataset at https://doi.org/10.1145/3242969.3242985.
